# Nanosized zinc oxide particles do not promote DHPN-induced lung carcinogenesis but cause reversible epithelial hyperplasia of terminal bronchioles

**DOI:** 10.1007/s00204-013-1086-5

**Published:** 2013-07-06

**Authors:** Jiegou Xu, Mitsuru Futakuchi, David B. Alexander, Katsumi Fukamachi, Takamasa Numano, Masumi Suzui, Hideo Shimizu, Toyonori Omori, Jun Kanno, Akihiko Hirose, Hiroyuki Tsuda

**Affiliations:** 1 Laboratory of Nanotoxicology Project, Nagoya City University, 3-1 Tanabedohri Mizuho-ku, Nagoya, 467-8603 Japan; 2 Department of Molecular Toxicology, Nagoya City University Graduate School of Medical Sciences, 1-Kawasumi, Mizuho-cho, Mizuho-ku, Nagoya, 467-8601 Japan; 3 Core Laboratory, Nagoya City University Graduate School of Medical Sciences, 1-Kawasumi, Mizuho-cho, Mizuho-ku, Nagoya, 467-8601 Japan; 4 Department of Health Care Policy and Management, Nagoya City University Graduate School of Medical Sciences, 1-Kawasumi, Mizuho-cho, Mizuho-ku, Nagoya, 467-8601 Japan; 5 National Institute of Health Sciences, 1-18-1 Kamiyoga, Setagaya-ku, Tokyo, 158-8501 Japan

**Keywords:** Nanosized zinc oxide particles, Lung toxicity, Lung carcinogenesis, Epithelial hyperplasia of terminal bronchioles, Interstitial pneumonitis, Lung fibrosis

## Abstract

Zinc oxide (ZnO) is known to induce lung toxicity, including terminal bronchiolar epithelial hyperplasia, which gives rise to concerns that nanosized ZnO (nZnO) might lead to lung carcinogenesis. We studied the tumor promoting activity of nZnO by an initiation–promotion protocol using human c-Ha-*ras* proto-oncogene transgenic rats (H*ras*128 rats). The rats were given 0.2 % N-nitrosobis(2-hydroxypropyl)amine (DHPN) in the drinking water for 2 weeks and then treated with 0.5 ml of 250 or 500 μg/ml nZnO suspension by intra-pulmonary spraying once every 2 weeks for a total of 7 times. Treatment with nZnO particles did not promote DHPN-induced lung carcinogenesis. However, nZnO dose-dependently caused epithelial hyperplasia of terminal bronchioles (EHTB) and fibrosis-associated interstitial pneumonitis (FAIP) that were independent of DHPN treatment. Tracing the fate of EHTB lesions in wild-type rats indicated that the hyperplastic lesions almost completely disappeared within 12 weeks after the last nZnO treatment. Since nZnO particles were not found in the lung and ZnCl_2_ solution induced similar lung lesions and gene expression profiles, the observed lesions were most likely caused by dissolved Zn^2+^. In summary, nZnO did not promote carcinogenesis in the lung and induced EHTB and FAIP lesions that regressed rapidly, probably due to clearance of surplus Zn^2+^ from the lung.

**Electronic supplementary material:**

The online version of this article (doi:10.1007/s00204-013-1086-5) contains supplementary material, which is available to authorized users.

## Introduction

One of the most widely used nanomaterials is nZnO. The worldwide production of nZnO powder is increasing every year and was reported to have reached 1.4 million tons in 2011. It is used in rubber industry and electronics and in commercial products such as sunscreens and paints. In the biomedical field, it is used in baby powders, antiseptic ointments, and zinc oxide tapes to treat a variety of skin conditions (Baldwin et al. 2001; Hughes and McLean 1988). Recently, nZnO has gained interest in cancer applications or as an active anticancer drug (Rasmussen et al. 2010).

Micron or larger-sized ZnO particles are considered to be “Generally Recognized as Safe” (GRAS) in food additives by the FDA. However, exposure to fumes containing ZnO and other metal particles during welding or galvanizing processes is known to lead to metal fume fever (Antonini et al. 2003; Drinker and Fairhall 1933; Fine et al. 1997). Recent reports have shown that nZnO affects cell viability and induces reactive oxygen species (ROS) in many mammary cell types in tissue culture (Deng et al. 2009; Lee et al. 2008; Xia et al. 2008; Yang et al. 2009), cause proliferation of airway epithelial cells, goblet cell hyperplasia, interstitial pulmonary inflammation and fibrosis (Cho et al. 2011), and reversible inflammatory reaction in the bronchoalveolar lavage fluid in animal studies (Sayes et al. 2007; Warheit et al. 2009). nZnO also leads to DNA damage (Kermanizadeh et al. 2012) and micronuclei formation in vitro (Valdiglesias et al. 2013). While these in vitro and in vivo studies have provided some information on acute toxic effects of nZnO on certain cell types and animals, further in vivo studies are needed to determine whether nZnO has chronic toxic effects as in some other metal oxide particles. For example, epidemiological data indicate that exposures of aluminum oxide or iron oxide lead to pneumoconiosis in human (Hull and Abraham 2002; Sano 1963); titanium dioxide has carcinogenic activity in the rat lung (Heinrich et al. 1995; Xu et al. 2010). Such chronic toxicity data will have more impact on risk assessment of nZnO.

Since nZnO induces inflammatory reaction, ROS production, and genotoxicity, which are implicated in cancer development, in the present study, we tested the lung carcinogenicity of nZnO by an initiation–promotion protocol using human c-Ha-*ras* proto-oncogene transgenic (H*ras*128) rats, which have the same susceptibility to chemically induced lung carcinogenesis as their parent wild-type rats, but are highly susceptible to mammary tumor induction (Tsuda et al. 2005). The results indicated that nZnO did not have promotion effect on DHPN-induced lung and mammary carcinogenesis and caused reversible EHTB and FAIP.

## Materials and methods

### Animals

Forty-three female transgenic rats carrying the human c-Ha-RAS proto-oncogene (H*ras*128 rats) and 42 female wild-type Sprague–Dawley rats were obtained from CLEA Japan Co., Ltd. (Tokyo, Japan). The animals were housed in the Animal Center of Nagoya City University Medical School and maintained on a 12-h light/12-h dark cycle and received Oriental MF basal diet (Oriental Yeast Co. Ltd., Tokyo, Japan) and water ad libitum. The study was conducted according to the Guidelines for the Care and Use of Laboratory Animals of Nagoya City University Medical School, and the experimental protocol was approved by the Institutional Animal Care and Use Committee (H22M-19).

### Preparation, characterization of nZnO suspensions, and administration of nZnO to the lung

Zinc oxide particles (CAS No. 1314-13-2, MZ-500, without coating, with a mean primary diameter of 25 nm) were obtained from Tayca Cooperation, Osaka, Japan. The particles were suspended in 0.1 % Tween 20 saline at 250 or 500 μg/ml. The suspension was sonicated for 20 min to prevent aggregate formation.

Characterization of nZnO was conducted as follows: the shape of nZnO in the suspensions was imaged by transmission electron microscopy (TEM); element analysis was performed by an X-ray microanalyzer (EDAX, Tokyo, Japan), after aliquots of nZnO were loaded on a carbon sheet; the size distribution of nZnO in the 500 μg/ml suspension was analyzed using a Particle Size Distribution Analyzer (Shimadzu Techno-Research, Inc., Kyoto, Japan). The characterization results are shown in Figure S1.

Before being administrated to rats, the nZnO suspensions were further sonicated for 20 min. 0.5 ml of the nZnO suspensions was administrated to the lung by intra-pulmonary spraying (IPS) as described previously (Xu et al. 2010).

### Carcinogenicity study

The carcinogenic activity of nZnO was assessed in female H*ras*128 rats using an initiation–promotion protocol by which we used previously to evaluate lung and mammary carcinogenicity of titanium dioxide nanoparticles (Xu et al. 2010). Briefly, three groups of 10–11 female H*ras*128 rats aged 6 weeks were given 0.2 % DHPN (Wako Chemicals, Co., Ltd. Osaka, Japan) in the drinking water for 2 weeks, and Groups 4 and 5 (6 rats each) were given drinking water without DHPN. Two weeks later, Group 1 and Group 4 were administered 0.1 % Tween 20 saline, and Group 2, Group 3, and Group 5 were administered 250, 500, and 500 μg/ml nZnO suspensions by IPS once every two weeks from the end of week 4 to week 16, a total of 7 times. The total amounts of nZnO administered to Groups 1, 2, 3, 4, and 5 were 0, 0.875, 1.75, 0, and 1.75 mg/rat, respectively. The dosing was determined according to the permissible exposure limit for zinc oxide particles of the Occupational Safety and Health Administration (OSHA) (see Discussion). Three days after the last treatment, animals were killed and the organs (brain, lung, liver, spleen, kidney, mammary gland, ovaries, uterus, and neck lymph nodes) were fixed in 4 % paraformaldehyde in PBS buffer adjusted to pH 7.3 and processed for histological examination and transmission electron microscopy (TEM).

### Light microscopy, polarized light microscopy, and transmission electron microscopy

Hematoxylin–Eosin (H&E)-stained pathological slides of the lung and other major organs were used to observe nZnO with a light microscope and polarized light microscope (PLM) (Olympus BX51N-31P-O polarized light microscope, Tokyo, Japan) at 1,000× magnification. Localization of the illuminated particles was confirmed in the same H&E-stained sections after removing the polarizing filter.

Paraffin blocks were deparaffinized and embedded in epon resin and processed for nZnO observation and zinc element analysis, using a JEM-1010 transmission electron microscope (TEM) (JEOL, Co. Ltd, Tokyo, Japan) equipped with an X-ray microanalyzer (EDAX, Tokyo, Japan).

### Immunohistochemistry and Azan–Mallory staining

PCNA was detected using an anti-PCNA monoclonal antibody (Clone PC10, Dako Japan Inc., Tokyo, Japan). The antibody was diluted 1:200 in blocking solution and applied to deparaffinized slides, and the slides were incubated at 4 °C overnight. The slides were then incubated for 1 h with biotinylated species-specific secondary antibodies diluted 1:500 (Vector Laboratories, Burlingame, CA) and visualized using avidin-conjugated horseradish peroxidase complex (ABC kit, Vector Laboratories). To assess lung fibrosis, paraffin-embedded slides were deparaffinized, and collagen fibers were visualized by Azan–Mallory staining.

### Reversibility study and effects of ZnCl_2_ solution

To assess whether nZnO-induced terminal bronchiolar epithelial hyperplasia, interstitial pneumonitis, and lung fibrosis are reversible, we conducted reversibility experiments. Seven groups of 5 female wild-type Sprague–Dawley rats aged 10 weeks were administrated 0.5 ml of 0.1 % Tween 20 saline or 500 μg/ml nZnO suspension by IPS 2 times per week for 4 weeks. Group 1 was treated with 0.1 % Tween 20 saline and killed 1 day after the last IPS. Groups 2–7 were treated with 0.5 ml of 500 μg/ml nZnO suspension and killed at 1 day and 2, 4, 6, 8, and 12 weeks after the last IPS. For the comparison of the effects of zinc ion and nZnO, Group 8 was treated with 0.5 ml of 6.17 mM ZnCl_2_ solution (the molecular amount is equal to that of 500 μg/ml nZnO suspension) by IPS at the same frequency and time period as the nZnO groups and killed 1 day after the last IPS. The left lung was cut into pieces and frozen in liquid nitrogen for biochemical analysis, and the right lung was processed for histological examination. Other major organs were excised for histological examination, and the blood was collected for cytological and biochemical analysis.

### Gene expression analysis

The left lungs from Groups 1, 2, and 8 in the reversibility study described above were used for isolation of RNA. RNA was isolated by using TRizol reagent (Invitrogen of Life Technologies, CA).

For microarray analysis, 1 μg RNA from each rat of Group 1 was combined and 1 μg RNA from each rat of Group 2 was combined. The quality of the 2 mixtures of RNA samples was assessed and quantified using the Agilent 2100 BioAnalyzer RNA Nano chip system (Agilent Technologies, CA) prior to further manipulation. Microarray analysis was conducted by the 3-D Gene Chip (Toray Industries Inc., Kanagawa, Japan), and a total of 20,000 genes were analyzed. Microarray-based pathway analysis was performed by Toray Industries Inc., Kanagawa, Japan.

For reverse transcription-PCR (RT-PCR) and real-time PCR, first-strand cDNA synthesis from 1 μg of RNA was performed using SuperScript™ III First-Strand Synthesis System (Invitrogen of Life Technologies, CA) according to the manufacturer’s instructions. Primers are as follows: forward primer, 5′-TAGAATCGAGGTGCACAGGAGT-3′, reverse primer, 5′-TATTCCAGCAGGCTGTCAAAGA-3′, product size, 228 bp for Orm1; forward primer, 5′-AAGTGGAGGAGCAGCTGGAGTGG-3′, reverse primer, 5′-CCAAAGTAGACCTGCCCGGACTC-3′, product size, 155 bp for Tnfa, and forward primer, 5′-AGCCATGTACGTAGCCATCC-3′, reverse primer, 5′-CTCTCAGCTGTGGTGGTGAA-3′, product size, 228 bp for Actb. RT-PCR was conducted using an iCycler (BioRad Life Sciences, CA) as follows: 95 °C 20 s, 60 °C 20 s, 72 °C 30 s, 30 cycles for Orm1; 95 °C 20 s, 60 °C 20 s, 72 °C 20 s, 25 cycles for Tnfa, and 95 °C 20 s, 60 °C 20 s, 72 °C 30 s, 15 cycles for Actb. Real-time PCR analysis of Orm1 and Tnfa gene expression was performed with the 7300 real-time PCR system (Applied Biosystem, CA) using the premix reagent Power SYBR Green PCR Master Mix (Applied Biosystem, CA) according to the manufacturer’s instructions. The Actb gene was used as the normalizing reference gene.

### Determination of zinc ion

For detection of Zn^2+^ content in the lung tissue, 50–100 mg of the frozen lung tissues from the reversibility study described above were thawed at room temperature, rinsed with cold PBS 3 times, and homogenized for 30 s at the highest speed in 1 ml of T-PER, tissue protein extraction reagent (Pierce, Rockford, IL), with Polytron R PT 2100 homogenizer (Capitol Scientific Inc., TX). The homogenates were clarified by centrifugation at 10,000×*g* for 15 min at 4 °C, and the supernatants were used for Zn^2+^ detection. Zn^2+^ detection was performed using QuantiChrom™ Zinc Assay kit (BioAssay Systems, CA) according to the manufacturer’s instructions.

### In vitro nZnO dissolution assay

5 μl of 500 μg/ml nZnO suspension (2.5 μg/tube) and increasing amounts of 1 mg/ml human α 1 acid glycoprotein (Sigma-Aldrich, product number G9885) or bovine serum albumin (Sigma-Aldrich, product number A2058) were added to microtubes, and the total volume of each tube was adjusted to 100 μl with 0.1 % Tween 20 saline. The final protein concentration of human α 1 acid glycoprotein or bovine serum albumin was 0, 100, 200, 300, 400, and 500 μg/ml. The tubes were then incubated at 37 °C for 2 h. The nZnO particles were removed by centrifugation at 10,000×*g* for 5 min, and Zn^2+^ concentration in the supernatants was determined as described above.

### In vitro cytotoxicity assay

The induction and preparation of rat primary alveolar macrophages (PAM) has been described (Xu et al. 2010). 5 × 10^3^ PAMs, 1 × 10^3^ A549 cells (human lung adenocarcinoma cell line), and 2 × 10^3^ CCD34 cells (human lung fibroblast cell line) were seeded into 96-well culture plates and cultured overnight in 100 ml of RPMI 1,640 containing 10 % FBS. The cells were added with nZnO suspension or ZnCl2 solution to final concentrations of 0, 1, 5, or 25 μg/ml of nZnO and 0, 12.3, 61.7, or 308.6 nM of ZnCl2 (1, 5, and 25 μg/ml of nZnO are equal to 12.3, 61.7, and 308.6 nM of ZnCl2, respectively, in the amount of zinc element) and incubated for another 72 h. The cell viability was then determined using the Cell Counting Kit-8 (Dojindo Molecular Technologies, Rockville, MD) according to the manufacturer’s instruction.

### Statistical analysis

Statistical analysis was performed using ANOVA. Statistical significance was analyzed using a two-tailed Student’s *t*-test. A *p* value of <0.05 was considered to be significant.

## Results

### Carcinogenesis study in H*ras*128 rats

DHPN-induced lung alveolar cell hyperplasia and adenoma development was used for the end point observation to assess the carcinogenicity of nZnO in our medium-term assay. As shown in Table 1, the incidence and multiplicity (number/cm^2^ lung tissue section) of alveolar cell hyperplasia and adenoma in the groups treated with nZnO were not significantly different from the DHPN alone group. In the rats which received nZnO treatment without prior DHPN treatment, alveolar cell proliferation foci, recognized as thickening of the alveolar wall with proliferative alveolar epithelium, were observed, but significant differences from the saline group were not observed. In the mammary gland, significant inter-group difference in incidence and multiplicity of mammary tumors was also not observed (data not shown).

**Table 1:** Effect of nZnO on lung proliferative lesions in H128-*ras* rats

Treatment	No. of rats	DHPN-induced proliferative lesions						DHPN-independent EHTB^a^	
		ACH^a^		Ade^a^		ACH + Ade		Inc. (%)	Multiplicity^b^ (no./cm^2^ lung)
		Inc.^a^ (%)	Multiplicity^b^ (no./cm^2^ lung)	Inc. (%)	Multiplicity^b^ (no./cm^2^ lung)	Inc. (%)	Multiplicity^b^ (no./cm^2^ lung)		
DHPN + vehicle	11	11 (100)	2.43 ± 1.29	2 (18.1)	0.09 ± 0.20	11 (100)	2.52 ± 1.26	0 (0)	0
DHPN + 250 μg/ml nZnO	10	10 (100)	1.64 ± 1.09	3 (30.0)	0.12 ± 0.19	10 (100)	1.76 ± 1.03	7 (70)	0.86 ± 0.86 **
DHPN + 500 μg/ml nZnO	10	10 (100)	1.83 ± 1.05	5 (50.0)	0.30 ± 0.31	10 (100)	2.13 ± 1.23	10 (100)	4.93 ± 1.95 ***
Vehicle	6	0	0	0	0	0	0	0	0
500 μg/ml nZnO	6	2 (33.3)	0.16 ± 0.26	0	0	2 (33.3)	0.16 ± 0.26	6 (100)	3.91 ± 1.29 ***

^a^ACH, Ade, EHTB, and Inc. are abbreviations for alveolar cell hyperplasia, adenoma, epithelial hyperplasia of terminal bronchioles, and incidence, respectively

^b^Multiplicity is expressed as mean ± s.d

** and *** represent *p* values <0.01 and 0.001, respectively, versus DHPN + vehicle or vehicle

**Fig. 1:**
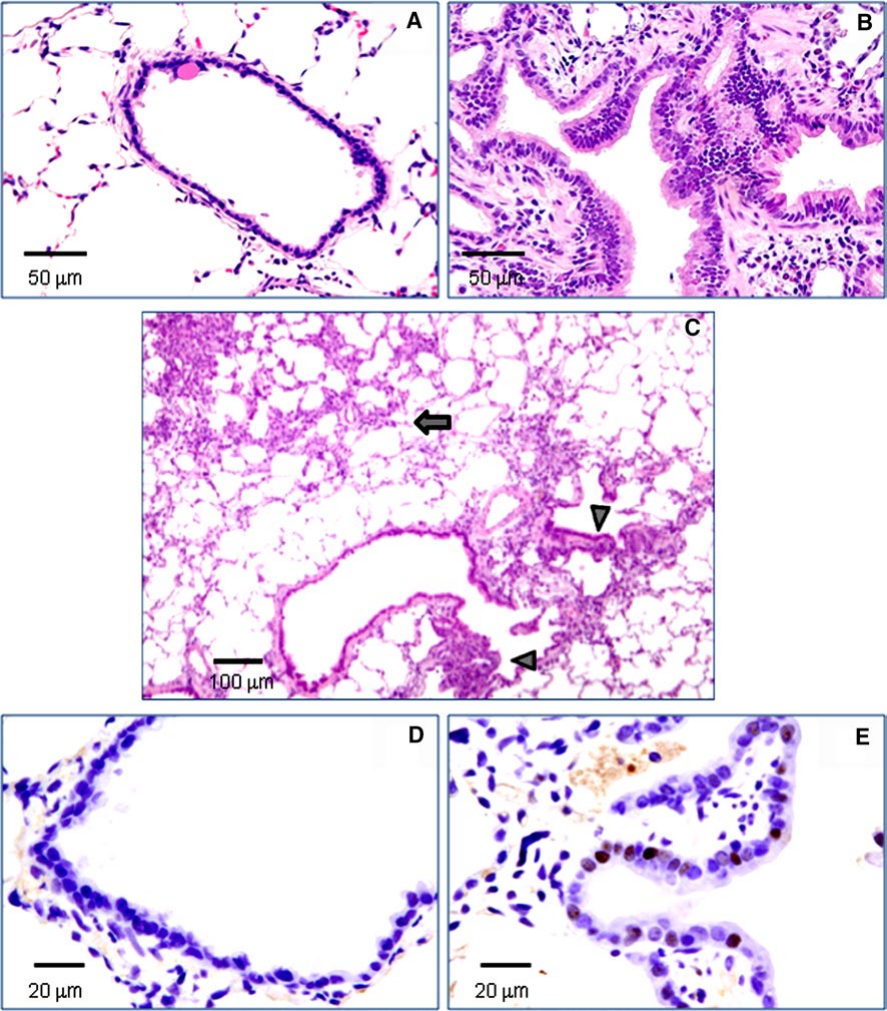
Induction of EHTB by nZnO. **a** representative normal terminal bronchiolar epithelium (NTBE); **b** EHTB in H&E-stained slides; **c** images and localizations of DHPN-induced alveolar hyperplasia (*arrow*) and nZnO-induced EHTB (*arrow heads*); **d** images of PCNA immunostaining in NTBE; and **e** in EHTB

A notable lesion induced in all the nZnO-treated groups was epithelial hyperplasia of terminal bronchioles (EHTB). The EHTB lesions had increased cell density, often with the epithelial cells arranged in 1–3 layers, and partly extended bronchiolar structures with transition to the normal terminal bronchioles (Fig. 1a, b). The EHTB lesions were independently localized from the DHPN-induced alveolar cell hyperplastic lesions (Fig. 1c). The incidences and multiplicity (number/cm^2^ lung tissue section) of EHTB in the groups treated with nZnO were significantly increased compared with that of the DHPN alone group. The increase was dose-dependent (Spearman rank correlation test, *p* < 0.001) (Table 1). Immunostaining with proliferating cell nuclear antigen (PCNA) indicated that proliferating bronchiolar epithelial cells were preferentially found in the EHTB lesions, but rarely found in the normal terminal bronchial epithelial areas (Fig. 1d, e).

**Fig. 2:**
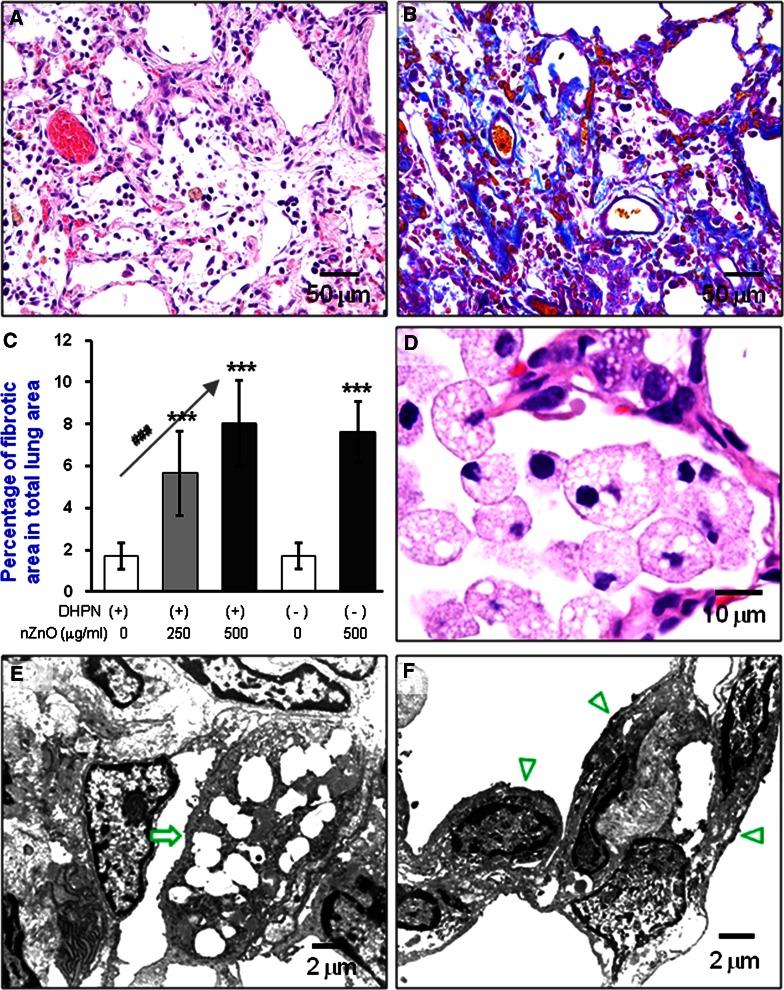
Induction of FAIP and observation of nZnO particles. **a** representative image of FAIP in rats treated with nZnO; **b** image of Azan–Mallory staining in the lung of rats treated with nZnO, showing collagen fibers; **c** percentage of the fibrotic area in total lung tissue area. ***<0.001 by two-tailed Student’s *t-*test versus the vehicle group; and ###*p* < 0.001 by Spearman rank correlation test. **d** image showing alveolar macrophages with vacuous phagocytosis vesicles; **e** and **f** TEM images showing alveolar macrophages (*arrow*) and epithelium (*arrow heads*), no nZnO particles being observed

Another lesion found in the groups treated with nZnO, and also independent of DHPN treatment, was interstitial pneumonitis (Fig. 2a). The lesion was usually associated with fibrosis of various thicknesses of the septal wall extruding into the alveolar structure (blue staining in Fig. 2b). Quantitative analysis indicated a significant increase in the fibrotic area in the rats treated with nZnO compared with that of rats treated with DHPN alone (Fig. 2c), and the increase was dose-dependent (Spearman rank correlation test, *p* < 0.001). In addition, the EHTB lesions often occurred near or within interstitial pneumonitis areas.

Light microscopic observation of the alveoli of the rats treated with nZnO showed infiltration of numerous macrophages mixed with a few neutrophils, eosinophils, and lymphocytes (data not shown). The nZnO particles were not found in any of the alveolar macrophages; these macrophages contained numerous vacuolar vesicles in the cytoplasm (Fig. 2d). Transmission electron microscopic (TEM) observation showed that nZnO particles were not found within the vacuolar vesicles (Fig. 2e) or in any alveolar tissue cells (Fig. 2f). The absence of a zinc peak was confirmed by elemental scanning with TEM-X-ray microanalysis (Figure S2). nZnO particles were also not detected under polarized light microscope observation. This feature was in contrast with titanium dioxide nanoparticles which were clearly observed in alveolar macrophages (Figure S3).

### Reversibility of EHTB and FAIP in wild-type rats

**Fig. 3:**
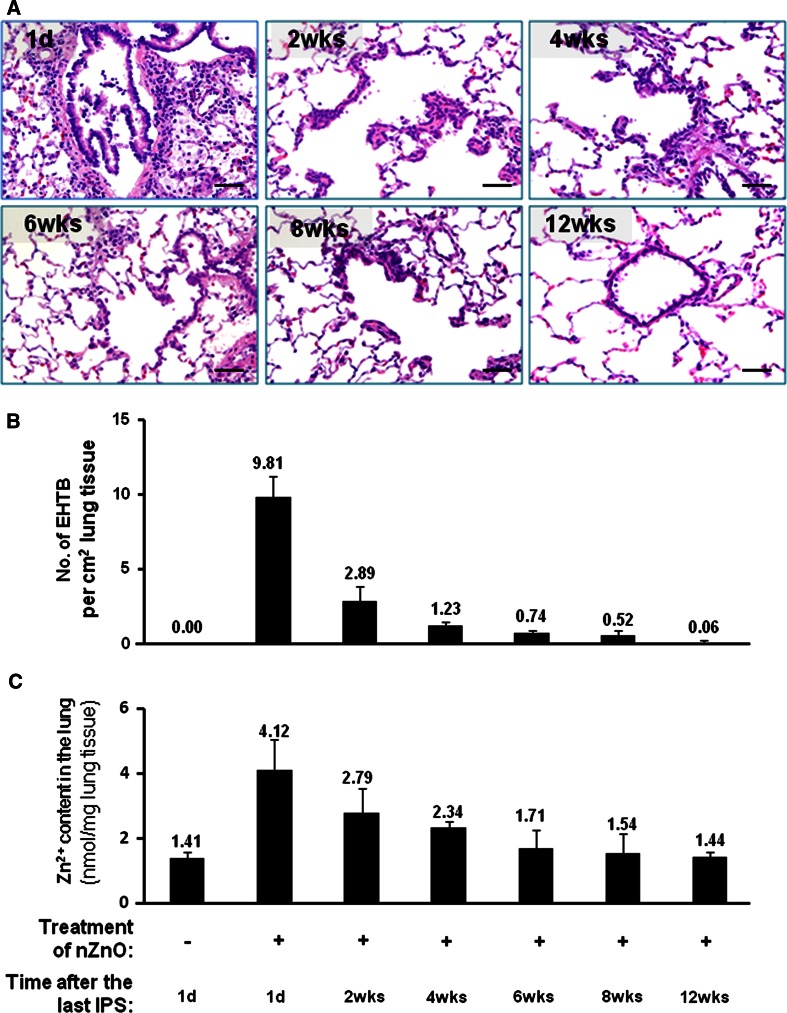
nZnO-induced EHTB and FAIP are reversible. Wild-type rats were treated with 500 μg/ml nZnO by IPS 2 times/week for 4 weeks and killed at different time points of 1 day (1d) and 2, 4, 6, 8, and 12 weeks (wks) after the last IPS. **a** histological images of the lung tissues; **b** number of EHTB per cm^2^ lung tissue and **c** Zn^2+^ content in the lung tissues at different time points. *Bars* = 50 μm

As in the H*ras*128 transgenic rats, nZnO induced EHTB and FAIP in wild-type Sprague–Dawley (SD) rats (Fig. 3a), and nZnO was not found in the lung tissue. nZnO-induced EHTB and FAIP gradually regressed with time (Fig. 3a), and the number of EHTB foci per square centimeter lung tissue section decreased from 9.81 ± 1.42 at day 1 to 0.06 ± 0.13 at week 12 after cessation of nZnO exposure (Fig. 3b). The total Zn^2+^ content in the lung tissue also gradually decreased (Fig. 3c) and was positively correlated with the number of EHTB (*r* = 0.96 by Pearson correlation test).

### Microarray analysis

Microarray analysis of the lung tissue indicated that nZnO treatment up-regulated the expression of 738 genes and down-regulated the expression of 267 genes (data not shown). The up-regulated inflammation-associated genes included chemotactic chemokines such as Cxcl5, Cxcl11, Ccl7, Cxcl2, Ccl2, and Cxcl1, proinflammatory cytokines such as Tnfa and Il6, and the acute-phase reactant Orm1 (Table S1). Pathway analysis showed an increase in inflammatory responses in which macrophages and TNFα play a central role (Figure S4). The gene expression profiling was consistent with the strong inflammatory responses in the lung observed by histological examination. Other pathways up-regulated by nZnO included classical complement activation pathway, matrix metalloproteinase pathway, cholesterol biosynthesis pathway and striated muscle contraction pathway, and treatment of nZnO down-regulated the adipogenesis pathway (data not shown).

### Effects of ZnCl_2_ solution on the lung of wild-type rats

**Fig. 4:**
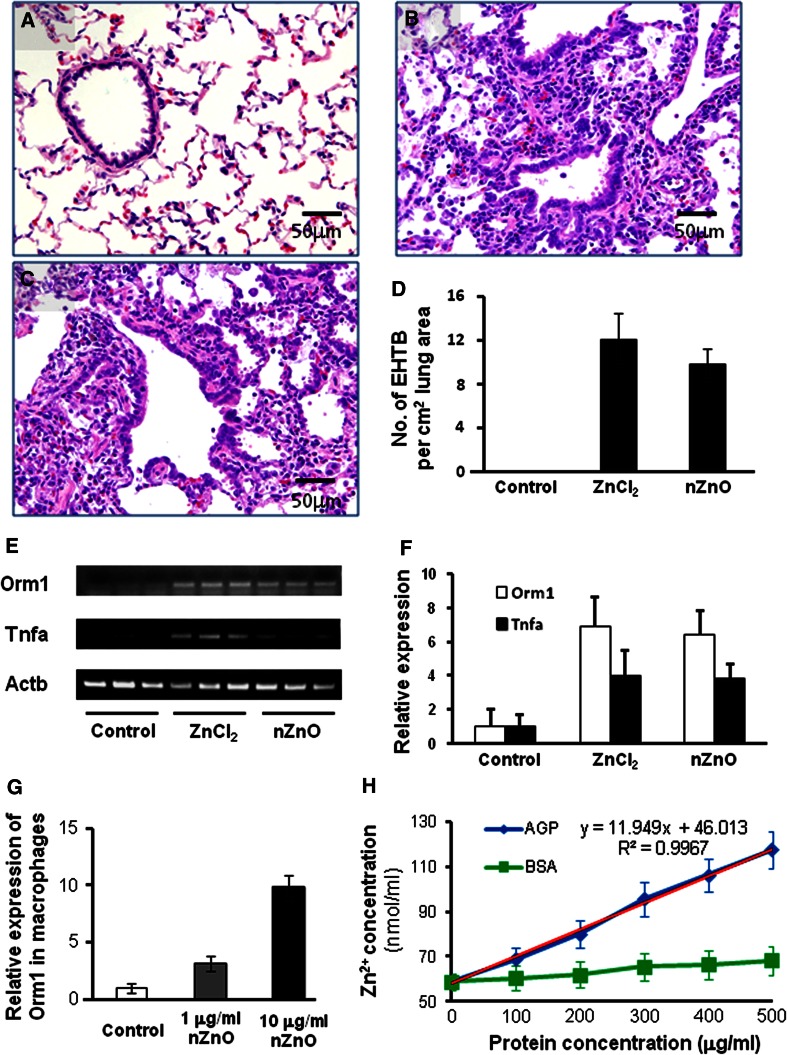
Similar effects of ZnCl_2_ solution and nZnO in induction of EHTB and FAIP in wild-type rats. **a** H&E-stained slides of the lungs of rats treated with vehicle; **b** with nZnO; and **c** with ZnCl_2_ solution, showing EHTB and FAIP; **d** comparable number of EHTB per square centimeter of the lung tissues induced by treatment of ZnCl_2_ and nZnO; **e** gene expression determined by RT-PCR of Orm1 and Tnfa, with Actb gene as an internal control; **f** real-time PCR analysis of gene expression of Orm1 and Tnfa, which was normalized with Actb expression; **g** induction of Orm1 expression in primary alveolar macrophages exposed to nZnO; and **h** effect of human alpha 1 acid glycoprotein (AGP) and bovine serum albumin (BSA) on dissolution of nZnO in vitro

To check whether the nZnO-induced EHTB and FAIP were due to dissolution of nZnO to Zn^2+^, we administered ZnCl_2_ solution (the molecular amount is equal to that of 500 μg/ml nZnO suspension) to the lung of rats by IPS. The lesions were histologically similar to those observed in the nZnO-treated rats (Fig. 4a, b, c). Quantitative analysis of EHTB indicated that the number of EHTB induced by ZnCl_2_ solution and nZnO was comparable (Fig. 4d).

To examine whether Zn^2+^ and nZnO have the same underlying molecular mechanisms, two genes, Tnfa and Orm1, which were determined to be up-regulated in the nZnO-treated rats by microarray analysis, were chosen for gene expression analysis. These genes were chosen because Tnfa-encoded tumor necrosis factor alpha is a multifunctional proinflammatory cytokine involved in a variety of acute and chronic inflammatory responses, and Orm1-encoded alpha 1 acid glycoprotein (AGP) is an acute-phase protein usually synthesized by hepatocytes in response to trauma, infection, and inflammation (Fournier et al. 2000). RT-PCR (Fig. 4e) and real-time PCR (Fig. 4f) showed that treatment with both ZnCl_2_ solution and nZnO increased the expression of Tnfa and Orm1 genes in the lung tissue, with a little higher induction in the ZnCl_2_ solution treated rats. Similarly, increased expression of Orm1 genes was found in primary alveolar macrophages exposed to nZnO in vitro (Fig. 4g). Interestingly, addition of human AGP to nZnO suspension dose-dependently promoted dissolution of nZnO from 59.1 nmol/ml (19.7 % dissolved, without addition of AGP) to 117.3 nmol/ml (39.1 % dissolved after addition of 500 μg/ml of AGP), while addition of bovine serum albumin (BSA) had little effect on dissolution of nZnO (Fig. 4h). Exposure of both nZnO and ZnCl_2_ solution resulted in dose-dependent cell death in vitro (Figure S5).

### Effects of nZnO particles and ZnCl_2_ solution on other organs and serum of wild-type rats

Obvious lesions and macrophages containing vacuolar vesicles were not found in other major organs including the liver, kidney, spleen, or brain by histological examination (data not shown). The results of blood cell examination are shown in Table S2: The only changes were increased proportions of monocytes and eosinophils that were rapidly recovered within 2 weeks post exposure. Biochemical examination of serum markers for tissue and organ injuries indicated no significant changes compared to the vehicle group (Table S3). Administration of nZnO or ZnCl_2_ to the lung led to a transient increase in serum Zn^2+^ concentration which returned to normal levels within 2 weeks after administration. The elevated serum Zn^2+^ did not affect the homeostasis of the other ions examined (Table S4).

## Discussion

In vivo nanomaterial toxicity usually implicates oxidative stress, inflammation (Nel et al. 2006), and other biological responses depending on the individual nanomaterial. In vitro assays related to carcinogenicity, such as mammalian cell transformation and gene mutation assays, cannot represent the complex in vivo processes of different biological alterations and are not always suitable for risk assessment of nanomaterial carcinogenicity. In the present study, we tested the carcinogenic activity of nZnO in H*ras*128 rats by an initiation–promotion protocol, by which we previously found promotion effect of nanosized titanium dioxide on DHPN-induced lung and mammary carcinogenesis (Xu et al. 2010). nZnO did not show any promotion effects on lung proliferative or neoplastic lesions, indicating that nZnO is not carcinogenetic. Also, nZnO did not promote DHPN-induced mammary carcinogenesis.

On the other hand, nZnO was found to induce EHTB in H*ras*128 rats and wild-type SD rats. EHTB is a proliferative lesion of the terminal bronchiolar epithelium. It should be noted that the localization of EHTB was independent from that of DHPN-induced alveolar cell hyperplasia. This observation clearly indicates that the DHPN-induced alveolar cell hyperplasia and EHTB have different etiology, the latter being induced by nZnO. We also observed 2 cases of alveolar cell hyperplasia out of 6 cases in the nZnO alone group. This is not significant and thus considered to be spontaneous or an inflammation-associated event. The EHTB lesions regressed when administration of nZnO was discontinued and completely disappeared after 12 weeks. Along with EHTB, the interstitial inflammatory changes often observed surrounding the EHTB lesions also regressed. Our data and other reports (Cho et al. 2011) indicate that the EHTB lesions do not progress directly to cancers but are reactive proliferation associated with inflammatory events. Similar reversible inflammatory changes in the bronchoalveolar lavage fluids by administration of nanoscale or fine ZnO particles via inhalation or intratracheal instillation have previously been reported (Warheit et al. 2009).

nZnO particles were not found in alveolar macrophages, in the lung tissue, or in other organs, suggesting that the particles were dissolved to Zn^2+^. Accordingly, we conducted experiments to determine whether Zn^2+^ would induce similar lesions. ZnCl_2_ solution induced closely similar lung lesions and gene expression profiles as nZnO, demonstrating that the observed lung lesions were caused by Zn^2+^. This was confirmed by increased Zn^2+^ level in the lung and serum after administration of nZnO. Interestingly, treatment with nZnO up-regulated the expression of the Omr1 gene in both the lung and the alveolar macrophages, and in vitro addition of Omr1-encoded AGP dose-dependently promoted nZnO dissolution. After Zn^2+^ was cleared from the lung, the EHTB and FAIP lesions disappeared, and this was evidenced by the positive correlation of EHTB number with Zn^2+^ content in the lung. Dissolution of nZnO has been reported to be particle size- and pH-dependent (Mudunkotuwa et al. 2012). Increased Omr1 expression possibly alters the microenvironment of the alveolar macrophages and the lung which accelerates nZnO dissolution. The elevated Zn^2+^ from nZnO dissolution possibly interferes with zinc ion homeostasis and leads to cytotoxic effects (Kao et al. 2012).

According to OSHA, the permissible exposure limit for zinc oxide particles is 15 mg/m^3^ of air for total dust and 5 mg/m^3^ for the respirable fraction (http://www.osha.gov/SLTC/healthguidelines/zincoxide/recognition.html). The inhalation exposure limit per kilogram of body weight per day for the respirable fraction is 192 μg, calculated from 6,000 ml of minute respiratory volume and 8 working hours for a 75 kg body weight worker. The dosing in the carcinogenesis study of the present study was approximately 35.5 and 71 μg/kg body weight a day (calculated from 125 to 250 μg every two weeks for a 250 g rat) and is lower than the OSHA limit for humans. Since nZnO has more potential to be ionized than larger ZnO particles because of its higher surface area (Mudunkotuwa et al. 2012), this feature should be taken into regulatory consideration.

It has been estimated that engineered nanomaterials will become a $1 trillion enterprise by 2015 (Nel et al. 2006), and ensuring health and environmental safety is a challenging task to the nanotechnology industry. Among numerous engineered nanomaterials, metal based or carbon based, most of which have been shown to have toxic effects to at least some extent, nZnO is a promising nanomaterial for biomedical applications. The results of the present study indicate that, although nZnO induced reversible lung toxicity, it did not cause carcinogenic or chronic progressive inflammatory lesions. Also, since it is biodegradable to ions, nZnO is easily cleared from the body (Rasmussen et al. 2010). Our study also suggests that the toxic effects of nZnO can be further decreased if efforts such as proper dosing and surface coating are made to lower the Zn^2+^ release from nZnO.

In conclusion, treatment of nZnO by IPS did not promote lung and mammary carcinogenesis in our carcinogenesis model. Although nZnO induced EHTB and FAIP, the lesions regressed rapidly along with clearance of surplus Zn^2+^ from the lung and serum. Thus, from a toxicological viewpoint, under the present experimental conditions, exposure of the lung to nZnO does not cause progressive neoplastic development or chronic fibrosis in the lung. These findings will be helpful in evaluating of the safety of nZnO used in biomedical applications, in which its use is of rather short duration, although long-term studies including inhalation studies are required to assess their occupational and environmental health hazards.

## Electronic supplementary material

Below is the link to the electronic supplementary material.

Supplementary material 1 (PDF 668 kb)
