# Supplementary material for: Nanosized zinc oxide particles do not promote DHPN-induced lung carcinogenesis but cause reversible epithelial hyperplasia of terminal bronchioles
Source: Arch Toxicol. 2013 Jul 6;88(1):65–75. doi: 10.1007/s00204-013-1086-5 (PMC3889829; doi:10.1007/s00204-013-1086-5)
Supplement: Supplementary file 1 — Supplementary material 1 (PDF 668 kb) [file 204_2013_1086_MOESM1_ESM.pdf]

Supplementary material

Nanosized zinc oxide particles do not promote DHPN-induced lung carcinogenesis but cause reversible epithelial hyperplasia of terminal bronchioles

Jiegou Xu[a, b], Mitsuru Futakuchi[b], David B. Alexander[a], Katsumi Fukamachi[b], Takamasa Numano[b], Masumi Suzui[b], Hideo Shimizu[c], Toyonori Omori[d], Jun Kanno[e], Akihiko Hirose[e] and Hiroyuki Tsuda[a,*]

[a] Laboratory of Nanotoxicology Project, Nagoya City University, 3-1 Tanabedohri, Mizuho-ku, Nagoya 467-8603, [b] Department of Molecular Toxicology, [c] Core Laboratory, [d] Department of Health Care Policy and Management, Nagoya City University Graduate School of Medical Sciences, 1-Kawasumi, Mizuho-cho, Mizuho-ku, Nagoya 467-8601, Japan, [e] National Institute of Health Sciences, 1-18-1 Kamiyoga, Setagaya-ku, Tokyo 158-8501, Japan.

[*] To whom correspondence should be addressed at Laboratory of Nanotoxicology Project, Nagoya City University, Phone: 81-52-836-3496, Fax: 81-52-836-3497, E-mail: htsuda@phar.nagoya-cu.ac.jp

The supplementary material contains 4 figures and 4 tables.

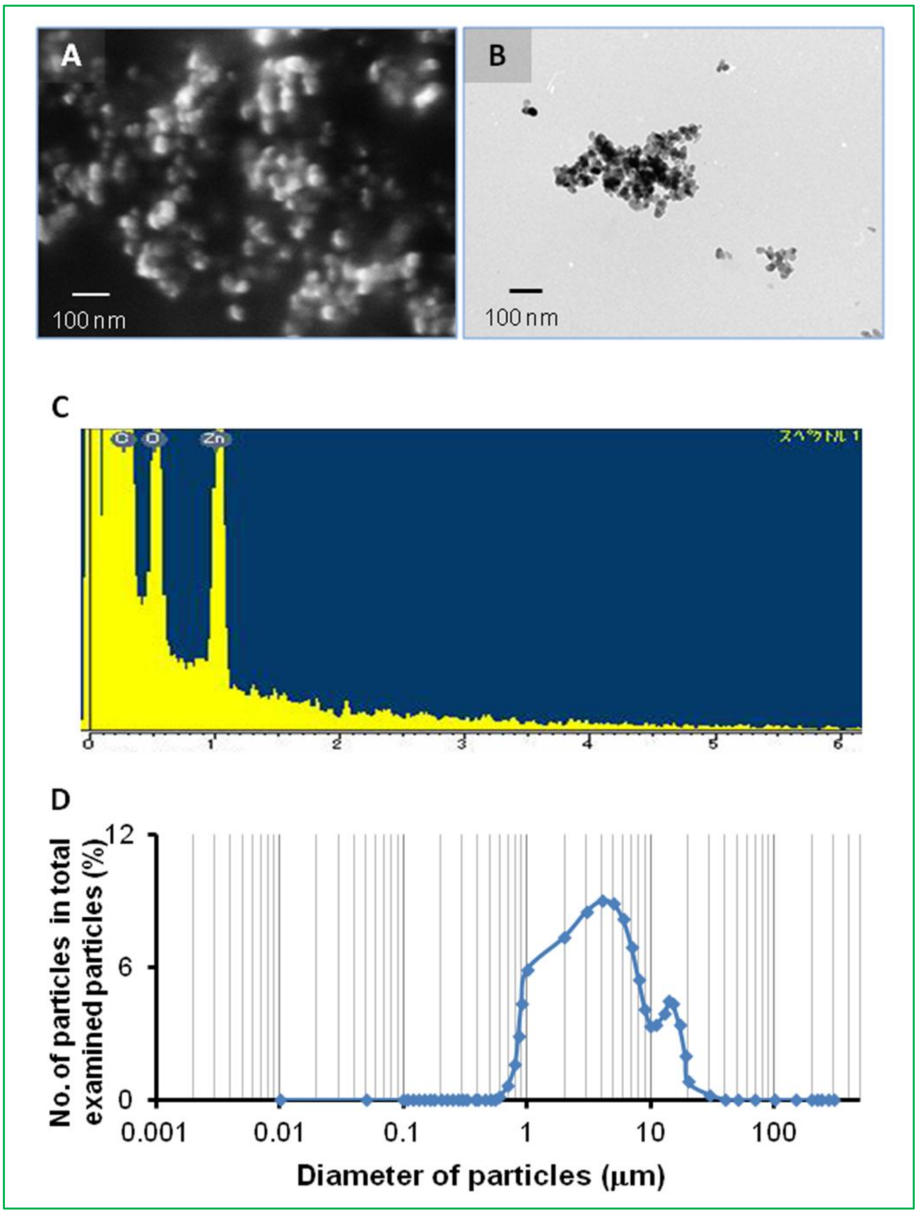

Figure S1. Characterization of nZnO in the suspensions

A and **B**, SEM and TEM images of nZnO; **C**, element scanning of nZnO by the X-ray microanalyzer EDAX connected with SEM, after aliquots of nZnO suspension were loaded on a carbon sheet; and **D**, size distribution of nZnO in the suspension. The median and average sizes were 2.039 µm and 1.994 ± 0.320 µm, indicating aggregate formation in the suspension.

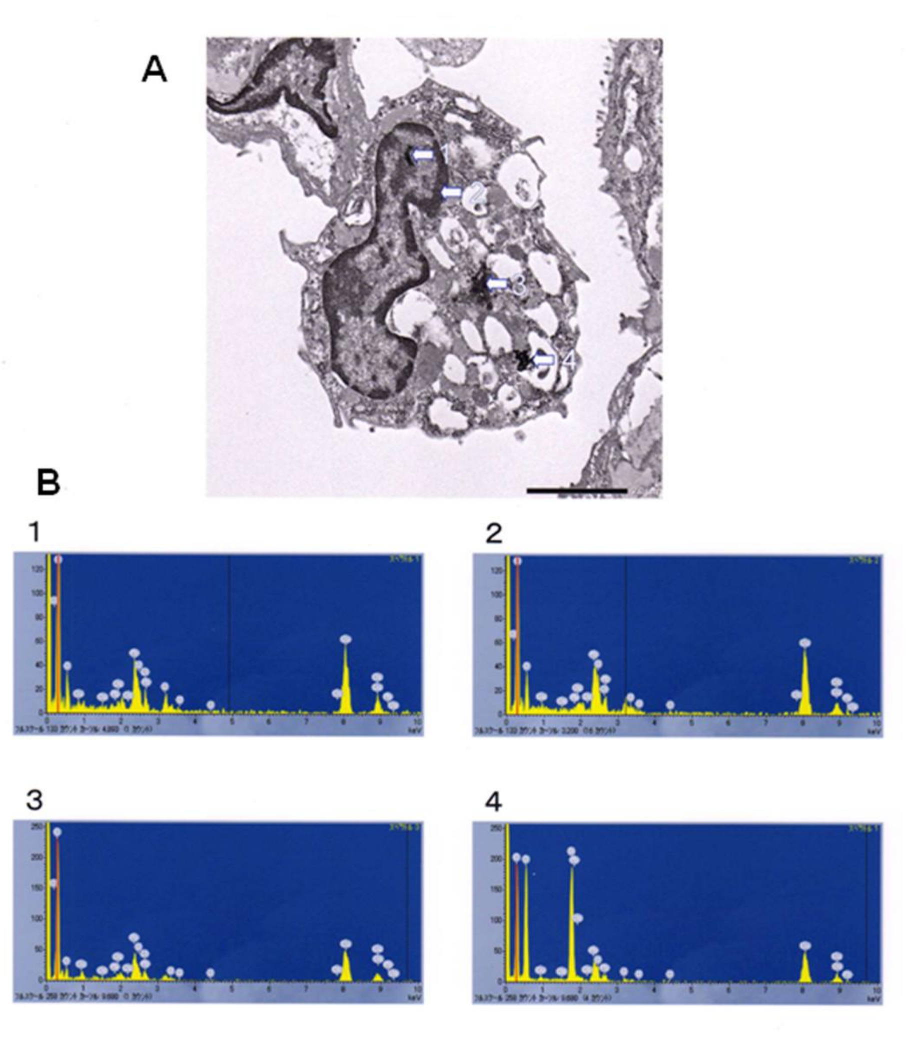

Figure S2. Zinc element was not detected in alveolar macrophages

A, TEM observation showed that an alveolar macrophage in the lung tissue of H*ras*128 rats treated with nZnO suspensions had vacant phagocytosis vacuoles in the cytoplasm. The bar=2μm. **B**, electron-dense parts (1, 2, 3 and 4 in **A**) were subject for element scanning by the X-ray microanalyzer EDAX. No zinc elemental peaks were observed.

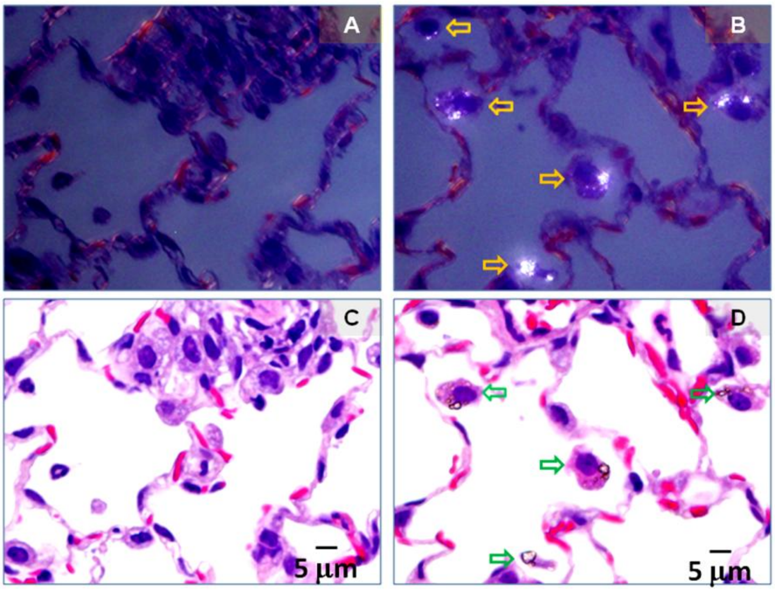

Figure S3. Polarized light microscope observation of nZnO in the lung

A, observation of H&E stained lung tissue slides from H*ras*128 rats treated with nZnO suspensions by polarized light microscope did not find any particles; **B**, similar observation of lung tissue slides from H*ras*128 rats treated with $nTiO_2$ suspensions from an earlier study [(1)] showed bright $nTiO_2$ particles; **C** and **D** are images of the same areas as in A and B, without the polarized filter.

Table S1. Inflammatory factors induced by nZnO in the rat lung

Gene	Fold increase	Function
**Cxcl5**	16.83	a potent chemotaxin involved in neutrophil activation.
**Cxcl11**	10.97	activated T-cell chemotactic protein
**Ccl7**	8.21	monocyte chemotactic protein
**Il24**	7.51	a member of the IL10 family induced during terminal differentiation in melanoma cells
**Cxcl2**	7.31	chemokine involved in the pulmonary inflammatory response
**Orm1**	6.86	Alpha 1 acid glycoprotein, an acute phase reactant involved in acute inflammation responses
**Ccl2**	6.67	implicated in the pathogenesis of diseases characterized by monocytic infiltrates
**Cxcl1**	5.11	a neutrophil chemoattractant
**Tnfa**	4.14	a multifunctional proinflammatory cytokine
**Ccl3**	3.68	monocyte and neutrophil chemotaxis
**Il6**	3.07	acute and chronic inflammation
**Ccl22**	2.97	chemoattractant for dendritic cells, natural killer (NK) cells, and Th2 subset of peripheral blood T cells
**Ifng**	2.86	a potent activator of macrophages
**Ccl17**	2.73	T-cell chemotactic and activation protein
**Csf2**	2.55	GM-CSF

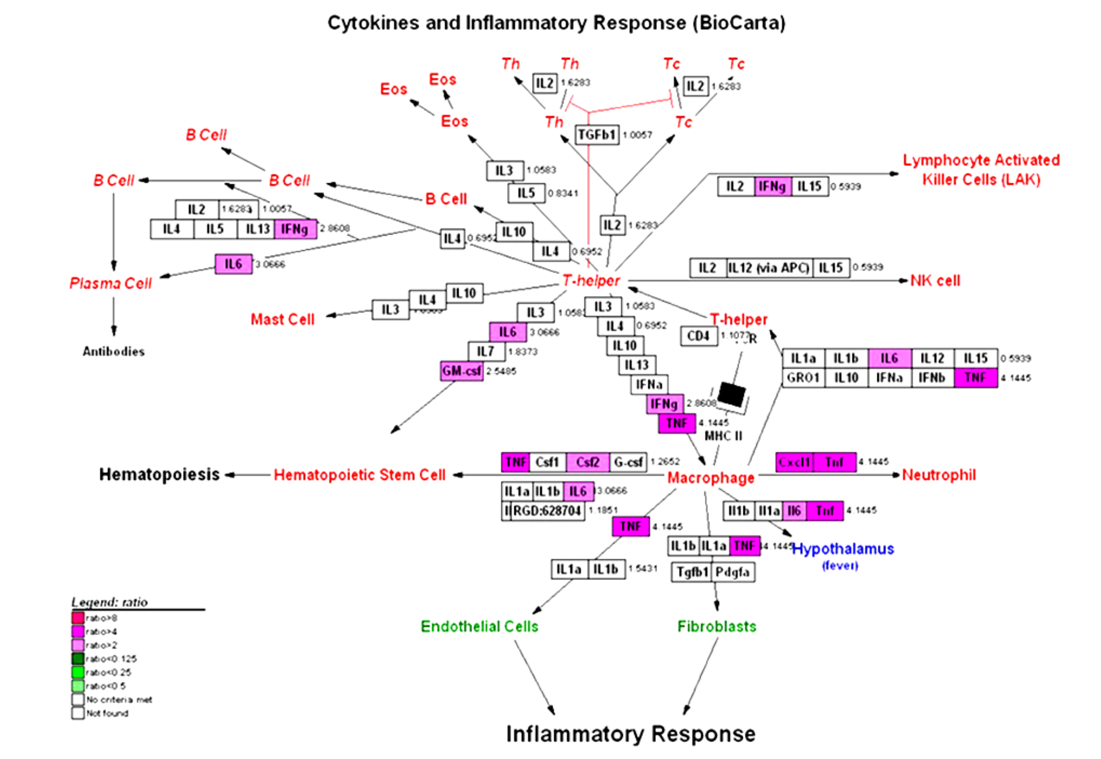

Figure S4. Enhanced inflammatory responses in the lung by nZnO

Inflammatory responses were analyzed by pathway analysis based on microarray data.

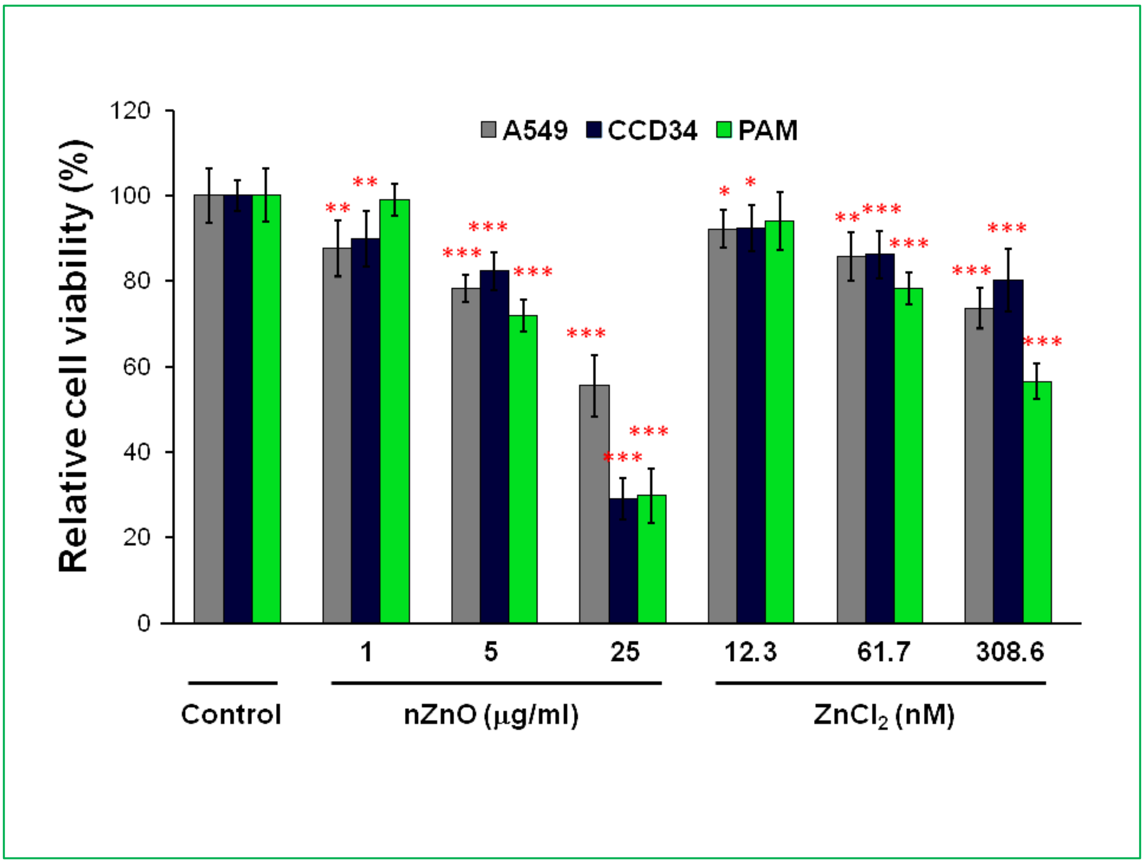

Figure S5. Cytotoxicity *in vitro*

nZnO suspension or ZnCl2 solution was added to A549, CCd34 or PAM cells. 1, 5 and 25 μg/ml of nZnO is equal to 12.3, 61.7 and 308.6 nM of $ZnCl_2$, respectively, in the amount of zinc element. n=6; *, ** and ***: $p<0.05$, 0.01 and 0.001, respectively.

Table S2. Blood cell examination

Treatment	Sacrifice	RBC (×10^4/μl)	HGB (g/dL)	Reticulocyte (%)	WBC (×10^2/μl)	Deferential leucocyte count (%)				
						Lymphocyte	Neutrophil	Monocyte	Eosinophil	Basophil
**0.1%TW20 saline**	1d	751.1 ±35.1	14.09 ±0.51	4.006 ±0.639	79.21 ±16.48	74.49 ±8.54	20.26 ±8.09	3.26 ±0.53	1.99 ±0.29	0
**500 μg/ml nZnO**	1d	735.3 ±24.3	13.99 ±0.35	4.681 ±0.850	83.48 ±17.96	67.50 ±8.23	24.64 ±8.20	4.25 ±0.71 *	3.61 ±1.42 *	0
**6.17 mM $ZnCl_2$**	1d	776.8 ±39.0	14.34 ±0.45	4.613 ±0.751	75.19 ±16.06	72.73 ±4.10	18.58 ±4.76	3.83 ±0.68	4.88 ±1.43 **	0
**500 μg/ml nZnO**	2 wks	770.3 ±34.2	14.39 ±0.44	4.916 ±0.568	76.64 ±16.66	75.61 ±4.39	19.16 ±4.80	2.90 ±0.53	2.33 ±0.29	0
**500 μg/ml nZnO**	4 wks	781.6 ±33.6	14.26 ±0.70	3.645 ±0.652	76.66 ±17.68	80.49 ±6.72	14.89 ±6.98	2.56 ±0.55	2.06 ±0.77	0
**500 μg/ml nZnO**	6 wks	785.1 ±32.4	14.08 ±0.48	4.173 ±0.444	67.55 ±21.49	69.85 ±7.01	25.50 ±6.56	2.74 ±0.54	1.91 ±0.70	0
**500 μg/ml nZnO**	8 wks	754.5 ±13.7	13.79 ±0.32	4.264 ±0.935	65.46 ±15.78	77.20 ±5.94	17.00 ±5.88	3.53 ±0.74	2.28 ±0.57	0
**500 μg/ml nZnO**	12 wks	772.6 ±34.2	14.06 ±0.53	3.731 ±0.744	45.98 ±13.12	75.36 ±7.05	18.44 ±6.85	3.26 ±0.79	2.95 ±1.18	0

Abbreviations: RBC, red blood cell; HGB, hemoglobin; and WBC, white blood cell. *, **: <0.05 and 0.01, respectively, by two tailed Student’s t-test.

Table S3. Serum biochemical examination

Treatment	Sacrifice	AST (U/L)	ALT (U/L)	ALP (U/L)	γ-GT (U/L)	CK (U/L)	LDH (U/L)	BUN (mg/dL)	CRE (mg/dL)	TP (g/dL)	ALB (g/dL)	A/G
**0.1%TW20 saline**	1d	85.6 ±15.5	32.6 ±3.8	342.5 ± 49.4	0.53 ±0.12	410.5 ± 158.0	889.6 ± 380.3	20.96 ± 3.61	0.384 ± 0.046	6.09 ±0.27	2.51 ±0.11	0.704 ± 0.015
**500 µg/ml nZnO**	1d	82.8 ±11.7	33.0 ±6.9	361.6 ± 85.4	0.48 ±0.26	391.4 ± 156.1	778.1 ± 272.9	19.43 ± 1.96	0.349 ± 0.024	6.16 ±0.41	2.53 ±0.24	0.695 ± 0.047
**6.17 mM $ZnCl_2$**	1d	75.8 ±8.5	31.3 ±4.5	448.3 ± 144.8	0.63 ±0.22	351.4 ± 152.6	714.0 ± 349.5	21.31 ± 1.47	0.343 ± 0.047	5.99 ±0.15	2.44 ±0.09	0.689 ± 0.020
**500 µg/ml nZnO**	2 wks	72.5 ±15.2	33.3 ±4.3	402.9 ± 113.2	0.73 ±0.20	391.1 ± 174.9	807.8 ± 434.8	22.65 ± 3.93	0.386 ± 0.033	6.53 ±0.07	2.74 ±0.14	0.724 ± 0.051
**500 µg/ml nZnO**	4 wks	72.6 ±8.2	36.3 ±7.3	385.1 ± 79.1	0.44 ±0.15	309.6 ± 59.2	624.6 ± 137.7	24.79 ± 6.54	0.346 ± 0.038	6.20 ±0.17	2.58 ±0.13	0.714 ± 0.077
**500 µg/ml nZnO**	6 wks	78.4 ±14.9	28.8 ±5.8	312.8 ± 116.1	0.51 ±0.16	317.4 ± 147.0	700.0 ± 298.7	22.30 ± 4.60	0.444 ± 0.029	6.39 ±0.34	2.65 ±0.14	0.710 ± 0.040
**500 µg/ml nZnO**	8 wks	90.5 ±18.7	34.3 ±4.8	289.4 ± 25.7	0.41 ±0.24	376.9 ± 144.1	719.3 ± 346.4	24.25 ± 2.52	0.379 ± 0.022	6.81 ±0.29	2.91 ±0.16	0.748 ± 0.042
**500 µg/ml nZnO**	12 wks	86.8 ±40.7	34.4 ±5.8	251.6 ± 97.8	0.57 ±0.27	329.2 ± 127.6	718.7 ± 287.2	20.85 ± 3.25	0.378 ± 0.043	6.64 ±0.45	2.87 ±0.21	0.763 ± 0.046

Abbreviations: AST, aspartate aminotransferase; ALT, alanine aminotransferase; ALP, alkaline phosphatase; γ-GT, gamma-glutamyl transpeptidase; CK, creatine kinase; LDH, lactate dehydrogenase; BUN, blood urea nitrogen; CRE, creatine; TP, total protein; ALB, albumin; and A/G, ratio of albumin to globulin.

Table S4. Serum ion examination

Treatment	Sacrifice	Zn^{2+} (μg/dL)	Ca^{2+} (mg/dL)	Mg^{2+} (mg/dL)	Na^{+} (mEq/L)	K^{+} (mEq/L)	Cl^{-} (mEq/L)
**0.1%TW20 saline**	1d	49.74 ±2.86	10.11 ±0.14	2.06 ±0.26	142.06 ±1.25	4.25 ±0.27	102.66 ±1.72
**500 μg/ml nZnO**	1d	54.49 ±2.17 *	10.23 ±0.27	2.03 ±0.17	142.23 ±0.52	4.36 ±0.37	102.73 ±1.09
**6.17 mM $ZnCl_2$**	1d	55.94 ±2.49 **	10.23 ±0.31	2.23 ±0.15	142.51 ±1.08	4.44 ±0.25	102.51 ±1.63
**500 μg/ml nZnO**	2 wks	50.81 ±3.91	10.38 ±0.26	2.18 ±0.18	142.64 ±0.68	4.17 ±0.21	103.38 ±1.46
**500 μg/ml nZnO**	4 wks	50.71 ±3.64	10.34 ±0.20	2.06 ±0.27	141.75 ±0.66	4.03 ±0.26	102.21 ±1.50
**500 μg/ml nZnO**	6 wks	50.22 ±2.60	10.03 ±0.17	2.00 ±0.13	142.54 ±1.51	3.96 ±0.19	104.65 ±1.20
**500 μg/ml nZnO**	8 wks	50.32 ±2.67	10.43 ±0.18	2.09 ±0.15	141.46 ±0.92	4.24 ±0.23	101.60 ±0.95
**500 μg/ml nZnO**	12 wks	50.03 ±2.81	10.28 ±0.37	2.04 ±0.15	145.18 ±0.78	4.18 ±0.22	106.67 ±1.72

*, **: <0.05 and 0.01, respectively, by two tailed Student's t-test.

1 Xu J, Futakuchi M, Iigo M et al. Involvement of macrophage inflammatory protein 1alpha (MIP1alpha) in promotion of rat lung and mammary carcinogenic activity of nanoscale titanium dioxide particles administered by intra-pulmonary spraying. Carcinogenesis 2010; 31(5): p. 927-35.
